# Is Mindfulness Linked to Life Satisfaction? Testing Savoring Positive Experiences and Gratitude as Mediators

**DOI:** 10.3389/fpsyg.2021.591103

**Published:** 2021-03-03

**Authors:** Rebecca Y. M. Cheung, Elsa Ngar-Sze Lau

**Affiliations:** ^1^Department of Early Childhood Education, Centre for Child and Family Science, Centre for Psychosocial Health, The Education University of Hong Kong, Hong Kong, Hong Kong; ^2^Department of Special Education and Counseling, Integrated Centre for Wellbeing, The Education University of Hong Kong, Tai Po, Hong Kong

**Keywords:** gratitude, mindfulness practitioners, life satisfaction, savoring positive experiences, mindfulness

## Abstract

Grounded in Mindfulness-to-Meaning Theory, this study examined the relation between dispositional mindfulness and life satisfaction through mediating mechanisms including savoring positive experiences and gratitude. A total of 133 Chinese mindfulness practitioners at 20–72 years old were recruited from a 3-day transnational meditation event in Hong Kong. Findings based on structural equation modeling indicated that controlling for sex, age, education, family income, number of hours of mindfulness practice per week, and type of administration, dispositional mindfulness was associated with satisfaction with life through savoring positive experiences and gratitude as mediators. The findings provided initial evidence for these processes between mindfulness and life satisfaction in the Chinese context. To promote life satisfaction, researchers and mental health practitioners should recognize the chain of mechanisms related to mindfulness.

## Introduction

Mindfulness refers to paying attention to the present moment non-judgmentally, including thoughts, emotions, and bodily sensations (Kabat-Zinn, 1994). It has been practiced in Buddhist and contemplative Christian communities to strengthen religiosity for centuries (Kabat-Zinn, 2011). Over the last few decades, mindfulness has been taught in secular programs to enhance people’s spirituality and improve mental health (Teasdale et al., 1995), regardless of faith traditions or religious orientations (Greeson et al., 2015). According to Mindfulness-to-Meaning Theory (Garland et al., 2015), mindfulness fosters decentering from distress to broadened attention and metacognitive awareness (Teasdale et al., 1995), which enhances emotion regulation, fosters positive emotions, and disengages people from autopilot. These processes further promote well-being, meaning in life, and better mental health (Brown and Ryan, 2003; Coffey and Hartman, 2008).

Indeed, recent studies have indicated emotion regulation as a mechanism between mindfulness and mental health (Desrosiers et al., 2013; MacDonald and Baxter, 2017; Cheung and Ng, 2019). Despite an enormous attention paid to the relations between mindfulness, down-regulating negative emotions, and psychological adjustment, relatively little has been done to investigate the up-regulation of positive emotions (Tugade and Fredrickson, 2007), such as savoring pleasant moments, thereby creating a gap in the literature. Returning to Mindfulness-to-Meaning theory, mindfulness broadens attention and awareness, such that people can recognize, appreciate, and savor pleasant moments to up-regulate positive emotions (see also Bryant, 1989). Supporting its theoretical tenets, Ritchie and Bryant (2012) found that university students’ dispositional mindful awareness was positively associated with different facets of savoring, including savoring the present moment, reminiscence, and anticipation. In another study, Wilson et al. (2020) showed that social support was associated with dispositional mindfulness, savoring, and self-compassion, all of which predicted psychological well-being, perceived stress, and depressive symptoms. More recently, Cheung and Ng (2020) showed that emerging adult’s mindful awareness longitudinally predicted savoring positive experiences *via* cognitive reappraisal, thereby providing incremental support for the effects of mindfulness on savoring.

As a “sister of mindfulness” (Rosenzweig, 2013), gratitude is closely related to mindfulness (Voci et al., 2019). Specifically, the broadened attention and awareness associated with mindfulness enhance emotion regulation and positive emotions including gratitude (Swickert et al., 2019). Mindful individuals are more likely to notice positive life experiences and be grateful for them (Emmons and Stern, 2013). In addition, their ability to non-judgmentally observe and be non-reactive to transient experiences fosters gratitude by virtue of deliberate and broadened attention, appreciation, and recognition of positive life experiences (Swickert et al., 2019). In a randomized controlled trial of an online 8-week mindfulness program, mindfulness practices enhanced gratitude at both immediate and one-month posttests (Ivtzan et al., 2016). Besides mindfulness, savoring positive experiences is also linked to feelings of gratitude. In an experimental study involving older adults (Bryant et al., 2020), savoring life lessons predicted life satisfaction through gratitude as a mediator. That is, savoring facilitates positive feelings of gratitude, which further enhances life satisfaction. As such, recent studies have highlighted the relations between mindfulness, savoring, and gratitude.

Despite the above findings, little has been done to examine both savoring and gratitude as mediating mechanisms between mindfulness and psychological outcomes, particularly in mindfulness practitioners. Being a key understudied research area (Wood et al., 2010), savoring positive experiences and feelings of gratitude may be important in giving rise to subjective well-being (Bryant et al., 2020). Although scholars have suggested close ties between mindfulness, savoring, and gratitude (McCullough, 2002; Bryant et al., 2011), relatively little has been done to substantiate the relations between them. Grounded in Garland et al.’s (2015) Mindfulness-to-Meaning Theory, the present study investigated savoring positive experiences and gratitude as mediators between dispositional mindfulness and life satisfaction among mindfulness practitioners.

## Method

### Participants

A total of 133 Chinese mindfulness practitioners (75.94% female) were recruited from a 3-day transnational meditation event in Hong Kong through a brief announcement and a booth at the event. Participants read and signed the consent form before they began the online (*n* = 39) or paper-and-pencil (*n* = 94) survey. The measures in the survey were not counter-balanced. Prior to the conduct of this study, ethics approval was sought at the authors’ university. Participants did not receive incentives for their participation.

Participants ranged from 20 to 72 years in age (*M* = 47.95; *SD* = 11.55), with a median family monthly income of HK$30,001–$40,000 (∼US$3,846.28–$5,141.39). Most participants had a bachelor’s degree or above (45.10%). Of the participants, 37.59% were single, 35.34% were married, 9.77% were divorced, and 17.30% were not otherwise specified. As for religion, 46.62% were self-identified Buddhists, 2.26% were Catholics, 3.76% were Protestants, 2.26% were non-Buddhist Chinese folk religion believers, 17.29% were Atheists, 8.27% did not have religious beliefs, and the rest were not otherwise specified (19.54%). Based on one-way ANOVA, the variables under study did not differ by religions, *p*s > 0.05. Independent samples *t*-test further revealed that the variables did not differ as a function of religious status (0 = non-religious; 1 = religious), *p*s > 0.05. Participants who did the survey online were older (*n* = *M*_*age*_ = 52.15; *SD*_*age*_ = 11.79) and reported less reminiscing (*M*_*reminiscing*_ = 2.70; *SD*_*reminiscing*_ = 0.62), i.e., an indicator of savoring, than did those who did the survey in paper-and-pencil format (*M*_*age*_ = 45.81; *SD*_*age*_ = 11.30; *M*_*reminiscing*_ = 3.18; *SD*_*reminiscing*_ = 0.56), *t*(95)*_*age*_* = 2.45, *p* < 0.05 and *t*(95)_*reminiscing*_ = −3.62, *p* < 0.001, respectively. Other variables did not differ by the type of administration, *p*s > 0.05. Participants had an average of 35.79 months of meditation practice (*SD* = 49.60), with 2.42 h of practice per week (*SD* = 2.89).

### Measures

#### Dispositional Mindfulness

The 20-item Five Facet Mindfulness Questionnaire-Short Form (FFMQ-SF; Hou et al., 2014) was used to assess dispositional mindfulness. A sample item included, “When I have distressing thoughts or images, I just notice them and let them go.” Participants rated on a 5-point scale from 1 (*never/very rarely true*) to 5 (*very often/always true*). Higher averaged scores indicated greater mindfulness. Cronbach’s alpha = 0.81.

#### Savoring Positive Experiences

The 24-item Savoring Beliefs Inventory (SBI; Bryant, 2003) was used to assess perceived beliefs of savoring on subscales including anticipation, savoring the moment, and reminiscing. Sample items included, “I feel a joy of anticipation when I think about upcoming good things” (anticipation), “I know how to make the most of good time” (savoring the moment), and “I can make myself feel good by remembering pleasant events from my past” (reminiscing). Participants rated on a 5-point scale from 1 (*strongly disagree*) to 5 (*strongly agree*). Item scores were averaged to form three subscale scores. Higher scores indicated a greater savoring tendency. In the preliminary analysis, the saturated measurement model showed significant factor loadings of the manifest variables on savoring (λ_*anticipation*_ = 0.85, λ_*savoring*the moment_ = 0.73, and λ_*reminiscing*_ = 0.86, *p*s < 0.001). Cronbach’s alphas for the subscales = 0.64, 0.77, and 0.72, respectively.

#### Gratitude

The 6-item Gratitude Questionnaire-6 (GQ-6; McCullough et al., 2002) was used to assess gratitude on a 7-point scale from 1 (*strongly disagree*) to 7 (*strongly agree*). A sample item included, “If I had to list everything that I felt grateful for, it would be a very long list.” Higher averaged scores indicated greater gratitude. Cronbach’s alpha = 0.58.

#### Life Satisfaction

Life satisfaction was assessed by the 5-item Satisfaction with Life Scale (SWLS; Diener et al., 1985) on a 7-point scale from 1 (*totally disagree*) to 7 (*totally agree*). A sample item included, “In most ways, my life is close to my ideal.” Higher averaged scores indicated greater life satisfaction. Cronbach’s alpha = 0.86.

### Data Analysis

Structural equation modeling was conducted using MPLUS, Version 8.3 (Muthén and Muthén, 2019) to investigate the mediating effects of savoring and gratitude between mindfulness and life satisfaction. Maximum likelihood method was used to evaluate the model fit to observed matrices of variance and covariance. Full information maximum likelihood estimation was used to handle missing data. Bootstrapping was used to evaluate the mediation effects.

## Results

**FIGURE 1:**
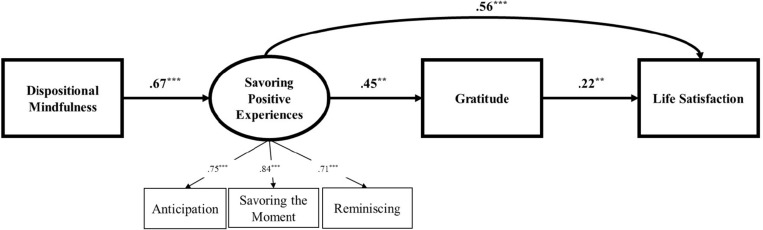
Savoring positive experiences and gratitude as mediators between mindfulness and life satisfaction among mindfulness practitioners. χ^2^(25) = 45.59, *p* = 0.01, CFI = 0.94, RMESA = 0.08, SRMR = 0.05. **p < 0.01, ***p < 0.001. Standardized coefficients are presented. Sex, age, education, income, hours of practice per week, and type of administration were included as covariates. Direct effects of mindfulness on gratitude and life satisfaction were not significant.

Table 1 shows the correlations, means, and *SD*s of the variables under study. Given the significant correlations between some variables hypothesized in the model and the background variables (i.e., participants’ sex, age, education, family income, hours of mindfulness practice per week, and type of administration), they were included as covariates in the structural equation model. The model fit adequately to the data, χ^2^(25) = 45.59, *p* = 0.01, CFI = 0.94, RMSEA = 0.08; SRMR = 0.05 (see Figure 1 and Table 2). In the measurement model, savoring positive experiences was significantly indicated by manifest variables including anticipation, savoring the moment, and reminiscing, *p*s < 0.001. In the structural model, mindfulness was positively associated with savoring positive experiences (β = 0.67, *p* < 0.001). Savoring was then related to gratitude (β = 0.45, *p* < 0.01). Savoring and gratitude were, in turn, related to life satisfaction (β = 0.56, *p* < 0.001 and β = 0.22, *p* < 0.01, respectively). As for the covariates, hours of mindfulness practice per week were related to greater dispositional mindfulness (*r* = 0.26, *p* < 0.01). Compared to being women, being men was related to more savoring (β = −0.19, *p* < 0.05) and less life satisfaction (β = 0.21, *p* < 0.01). Age (β = 0.27, *p* < 0.01), education (β = 0.19, *p* < 0.05), and type of administration (β = −0.14, *p* < 0.01) were related to life satisfaction, respectively. Income was not related to any variables (*p*s > 0.05).

**TABLE 1:** Zero-order correlations, means, ranges, and standard deviations of the variables.

**Variable**	**1.**	**2.**	**3.**	**4.**	**5.**	**6.**	**7.**	**8.**	**9.**	**10.**	**11.**	**12.**
1. Sex (0 = male; 1 = female)	–	–										
2. Age	−0.05	–										
3. Formal education (years)	−0.001	−0.37***	–									
4. Income (in Hong Kong Dollars)	0.06	0.02	0.20*	–								
5. Hours of practice per week	−0.02	0.13	−0.09	−0.09	–							
6. Type of administration (0 = paper; 1 = online)	−0.17	−0.27**	0.18	0.11	0.08	–						
7. Dispositional mindfulness	−0.16	0.19*	−0.01	0.08	0.27**	0.11	–					
8. Savoring: Anticipation	−0.24*	−0.12	0.05	0.11	−0.05	0.10	0.44***	–				
9. Savoring: Savoring the moment	−0.22*	0.02	0.07	0.11	0.10	0.07	0.57***	0.60***	–			
10. Savoring: Reminiscing	−0.24*	−0.19	0.21*	0.22*	−0.03	0.33***	0.38***	0.71***	0.61***	–		
11. Gratitude	−0.25**	−0.13	−0.08	0.11	−0.02	−0.05	0.28**	0.41***	0.33***	0.35***	–	
12. Life Satisfaction	−0.02	0.23*	0.12	0.22*	0.08	−0.10	0.47***	0.45***	0.56***	0.31**	0.45***	–
*M*	–	47.95	14.62	40892.86	2.42	–	3.23	2.90	2.98	3.02	5.45	4.78
*SD*	–	11.55	3.10	28555.42	2.89	–	0.50	0.59	0.65	0.61	0.79	1.30
Range	–	20–72	6–21	5,000–105,000	0–15	–	2.05–4.50	1.86–5.00	1.50–5.00	1.63–5.00	1.00–7.00	2.83–7.60

*p < 0.05, **p < 0.01, and ***p < 0.001.

**TABLE 2:** Path estimates of the mediation model under study.

**Parameter**	**Unstandardized B (*SE*)**	**Standardized β**
**Measurement model**		
**Savoring positive experiences**		
→ Anticipation	1.00^*f*^	0.75***
→ Savoring the moment	1.25 (0.17)	0.84***
→ Reminiscing	0.99 (0.11)	0.71***

**Structural model**		
**Dispositional mindfulness**		
→ Savoring positive experiences	0.59 (0.09)	0.67***
→ Gratitude	−0.10 (0.22)	−0.06
→ Life satisfaction	0.07 (0.29)	0.03
**Savoring positive experiences**		
→ Gratitude	0.81 (0.28)	0.45**
→ Life satisfaction	1.65 (0.42)	0.56***
**Gratitude**		
→ Life satisfaction	0.36 (0.14)	0.22**
**Sex (0 = male; 1 = female)**		
↔ Dispositional mindfulness	−0.02 (0.01)	−0.14
→ Savoring positive experiences	−0.28 (0.13)	−0.19*
→ Gratitude	−0.28 (0.26)	−0.11
→ Life satisfaction	0.93 (0.32)	0.21**
**Age**		
↔ Dispositional mindfulness	1.00 (0.53)	0.17
→ Savoring positive experiences	−0.01 (0.00)	−0.19
→ Gratitude	0.01 (0.01)	0.20
→ Life satisfaction	0.03 (0.01)	0.27**
**Education**		
↔ Dispositional mindfulness	−0.01 (0.14)	−0.01
→ Savoring positive experiences	0.00 (0.01)	0.02
→ Gratitude	−0.02 (0.03)	−0.07
→ Life satisfaction	0.08 (0.03)	0.19*
**Income**		
↔ Dispositional mindfulness	0.16 (0.13)	0.11
→ Savoring positive experiences	0.02 (0.01)	0.10
→ Gratitude	0.02 (0.03)	0.07
→ Life satisfaction	0.03 (0.03)	0.07
**Hours of practice per week**		
↔ Dispositional mindfulness	0.37 (0.14)	0.26**
→ Savoring positive experiences	−0.02 (0.01)	−0.11
→ Gratitude	−0.01 (0.03)	−0.05
→ Life satisfaction	0.03 (0.03)	0.07
**Type of administration (0 = online; 1 = paper)**		
↔ Dispositional mindfulness	0.03 (0.02)	0.11
→ Savoring positive experiences	−0.03 (0.08)	−0.03
→ Gratitude	0.10 (0.15)	0.06
→ Life satisfaction	−0.41 (0.20)	−0.14*

*p < 0.05, **p < 0.01, and ***p < 0.001.

The total indirect effect from mindfulness to life satisfaction *via* savoring positive experiences and gratitude was significant (β = 0.43, *p* < 0.01). Based on 5,000 bootstrap samples with replacement, the 95% confidence interval (CI) indicated that the standardized indirect effect between mindfulness and life satisfaction did not include a zero, CI: (0.22, 0.76) thereby suggesting savoring and gratitude as mediating processes.

### Supplementary Analysis

A second model was conducted with reversed order between savoring and gratitude, as predicted by mindfulness. The model fit adequately to the data, χ^2^(25) = 45.59, *p* = 0.01, CFI = 0.94, RMSEA = 0.08; SRMR = 0.05. Mindfulness was associated with gratitude (β = 0.24, *p* < 0.01). Gratitude was then related to savoring (β = 0.27, *p* < 0.01). Gratitude and savoring were, in turn, related to life satisfaction (β = 0.22, *p* < 0.01 and β = 0.56, *p* < 0.001, respectively). The 95% CI indicated that the standardized indirect effect between mindfulness and life satisfaction did not include a zero, CI: (0.22, 0.76), thereby suggesting gratitude and savoring as mediating processes.

## Discussion

Supporting Mindfulness-to-Meaning Theory (Garland et al., 2015), this study evidenced savoring positive experiences and feelings of gratitude as mediating processes between mindfulness and life satisfaction above and beyond the effects of sex, age, education, family income, number of hours of mindfulness practice per week, and type of administration. In a sample of mindfulness practitioners, this study showed that dispositional mindfulness was related to greater savoring positive experiences, i.e., an ability to up-regulate positive emotions. Savoring was, in turn, associated with more gratitude, which was then related to greater life satisfaction. These findings add to the growing evidence of the mechanisms between mindfulness and psychological adjustment (Brown and Ryan, 2003; Desrosiers et al., 2013; Cheung and Ng, 2019; Cheung et al., 2020).

By mindfully orienting to the present moment through broadened awareness, non-judgmental acceptance, and disengagement from autopilot, mindful individuals were more capable of savoring positive experiences, which fostered their feelings of gratitude. These findings corroborated previous research that showed the link between mindfulness and subjective well-being (MacDonald and Baxter, 2017). The study also indicated that the process of savoring, such as feeling joyful from looking forward, making the most of good times, and recalling happy moments, could enhance feelings of gratitude (Bryant et al., 2011, 2020). That is, as a “sister of mindfulness” (Rosenzweig, 2013), not only was gratitude associated with dispositional mindfulness, but the relation was supported through the underlying mechanism of savoring positive experiences. Supplementary analysis further revealed that mindfulness fostered savoring positive experiences through gratitude. That is, mindful individuals were more likely to notice positive life experiences and be grateful for them (Emmons and Stern, 2013; Swickert et al., 2019). With more feelings of gratitude, individuals then had more positive experiences to savor, reminisce, and look forward to. Based on the mixed findings, future longitudinal studies are necessary to establish the temporal sequence between gratitude and savoring.

As for the covariates, the number of hours of mindfulness practice per week was associated with greater dispositional mindfulness in this study. Our model also echoes with previous findings that age was negatively associated with savoring (Palmer and Gentzler, 2019) and positively associated with life satisfaction (Hong and Giannakopoulos, 1994). Contrary to previous studies showing that women had a greater savoring tendency (Bryant et al., 2011; Kim and Bryant, 2017), our data suggested that men were more likely to savor. However, in this study men were also more likely to report lower life satisfaction. Given that our sample was 75.94% female, future studies should collect a balanced sample of men and women to rule out or verify the effect of sex on savoring and life satisfaction. Finally, although income was not related to any variables, both age and education were related to better life satisfaction (see also Horley and Lavery, 1995; Yakovlev and Leguizamon, 2012). Likewise, type of administration was linked to better life satisfaction. Therefore, future studies should examine how different background variables may affect psychological wellness.

This study provided support to suggest savoring and gratitude as mediators between mindfulness and life satisfaction. Nevertheless, it is important to also note its limitations. First, although the Mindfulness-to-Meaning Theory guided the hypothesized model, we did not include several variables covered by the theory, including positive reappraisal, prosocial actions, and purpose in life due to feasibility issues. Future studies should incorporate these variables to fully test the theory. Second, this study utilized self-report measures. Future research should use multiple methods and reporters to reduce biases. Third, the cross-sectional design precluded us from drawing conclusions on causal effects and temporal sequence between variables. Relatedly, the supplementary analysis with reversed direction of effects yielded similar findings compared to the proposed model. Therefore, longitudinal studies are needed to further distinguish the directionality of effects between savoring and gratitude. Experimental designs are also necessary to verify the causal effects. Fourth, the Cronbach’s alpha of GQ-6 (McCullough et al., 2002) and SBI (Bryant, 2003) was low. Thus, findings must be interpreted with caution. Fifth, we recruited mindfulness practitioners from a 3-day transnational meditation event through a brief announcement and a small booth. It was not feasible for us to draw conclusions on the response rate due to our insufficient data about the event. In addition, our sample size was small. Most of the participants were women, Buddhists, and with a relatively high socioeconomic status (see also Census and Statistics Department, 2020). To rule out self-selection biases and draw conclusions on the generalizability of the findings, population-based studies with larger samples should be conducted to document the response rate and increase generalizability of the findings. Next, although the variables under study did not differ by religions or religious status, they may be linked to religiosity and spirituality. Future studies should further examine how religiosity and spirituality are associated with mechanisms of mindfulness and well-being. Finally, in terms of survey administration, people who participated online were older and reported less reminiscing. Therefore, future studies should examine why and how the type of administration may affect participants’ responses.

Despite the limitations, the present study lends support to Mindfulness-to-Meaning Theory (Garland et al., 2015) and broadens the literature on the mediating role of savoring and gratitude between mindfulness and life satisfaction among mindfulness practitioners. While experimental research is needed to verify specific causal and directionality of effects, our findings indicated that mental health practitioners should be aware of a potential chain of mechanisms between mindfulness and life satisfaction, including savoring positive experiences and feelings of gratitude. As for research implications, randomized controlled trials, experimental research, and longitudinal and translational research involving mindfulness practice merit future investigation.

## Data Availability Statement

The dataset analyzed in this article is not publicly available. Requests to access the dataset should be directed to RC, rymcheung@eduhk.hk.

## Ethics Statement

The studies involving human participants were reviewed and approved by Human Research Ethics Committee, The Education University of Hong Kong. The participants provided their written informed consent to participate in this study.

## Author Contributions

RC contributed to conceptualization, methodology, formal analysis of this study, and writing of the manuscript. EN-SL contributed to conceptualization of this study and revision of the manuscript. Both authors were accountable for the final version of the manuscript.

## Conflict of Interest

The authors declare that the research was conducted in the absence of any commercial or financial relationships that could be construed as a potential conflict of interest.
